# Addition of eptifibatide and manual thrombus aspiration to ticagrelor does not improve long-term survival after STEMI treated with primary PCI

**DOI:** 10.3389/fphar.2024.1415025

**Published:** 2024-06-13

**Authors:** Paul-Adrian Călburean, Paul Grebenișan, Ioana-Andreea Nistor, Krisztina Pal, Victor Vacariu, Reka-Katalin Drincal, Alissa Anamaria Ion, István Adorján, Tiberiu Oltean, László Hadadi

**Affiliations:** ^1^ Department of Medical Informatics and Biostatistics, University of Medicine, Pharmacy, Science and Technology “George Emil Palade” of Târgu Mureş, Târgu Mureş, Romania; ^2^ Emergency Institute for Cardiovascular Diseases and Transplantation Târgu Mureş, Târgu Mureş, Romania

**Keywords:** ST-segment elevation myocardial infarction, ticagrelor, eptifibatide, manual thrombus aspiration, acute coronary syndrome

## Abstract

**Background:** Current guidelines recommend that glycoprotein IIb/IIIa inhibitor (GPI) and manual aspiration thrombectomy should not be routinely used in patients with ST-segment elevation myocardial infarction (STEMI) treated by primary percutaneous coronary intervention (pPCI), although there is a lack of dedicated studies. The aim of this study was to examine the impact of combined usage of a potent P2Y12 inhibitor, GPI, and manual aspiration thrombectomy on long-term survival after STEMI.

**Methods:** All STEMI patients treated by pPCI in a tertiary center who have been included prospectively in the local PCI registry between January 2016 and December 2022 were analyzed in this study. Patients were excluded if they required oral anticoagulation or bridging between clopidogrel or ticagrelor during hospitalization.

**Results:** A total of 1,210 patients were included in the present study, with a median follow-up of 2.78 (1.00–4.88) years. Ticagrelor significantly reduced all-cause and cardiovascular-cause mortality [HR = 0.27 (0.21–0.34), *p* < 0.0001 and HR = 0.23 (0.17–0.30), *p* < 0.0001, respectively]. Eptifibatide significantly reduced all-cause and cardiovascular-cause mortality [HR = 0.72 (0.57–0.92), *p* = 0.002, and HR = 0.68 (0.52–0.89), *p* = 0.001, respectively]. Manual thrombus aspiration had no significant effect on both all-cause and cardiovascular-cause mortality. In multivariate Cox regression, all-cause mortality was reduced by ticagrelor, while eptifibatide or manual thrombus aspiration had no significant effect. However, cardiovascular-cause mortality was reduced by both ticagrelor and eptifibatide, while manual thrombus aspiration had no significant effect.

**Conclusion:** Ticagrelor consistently reduced cardiovascular and all-cause mortality, while eptifibatide reduced only cardiovascular mortality. Manual thrombus aspiration provided no long-term benefit. Our findings support the current guideline recommendation that GPI and manual aspiration thrombectomy should not be routinely used in treatment of STEMI with pPCI.

## Introduction

ST-segment elevation myocardial infarction (STEMI) is a common acute manifestation of atherosclerotic cardiovascular disease with high rates of long-term mortality, even in the context of primary percutaneous coronary intervention (primary PCI), which is the recommended myocardial reperfusion therapy (Byrne et al., 2023). Numerous strategies for improving outcomes in treatment of STEMI with primary PCI have been proposed and implemented, including implementation of nationwide STEMI networks, minimizing door-to-balloon time, and optimizing antithrombotic strategies (Byrne et al., 2023). Regarding the latter, current clinical practice guidelines for management of acute coronary syndrome (ACS) recommends an antithrombotic treatment with dual antiplatelet therapy (DAPT) with aspirin and a potent P2Y12 receptor inhibitor (ticagrelor or prasugrel instead of clopidogrel) and parenteral anticoagulation with unfractionated heparin (Byrne et al., 2023). A more intensive antithrombotic treatment with GP IIb/IIIa receptor inhibitors and/or manual aspiration thrombectomy is not recommended to be routinely used, but only in selected cases with high thrombotic burden or no-reflow phenomenon as a bail-out strategy (Byrne et al., 2023). As there are no dedicated studies underlying this specific recommendation, it has led some authors to question if it is not a rather defensive approach in treating STEMI patients (Rakowski et al., 2023). In the context of numerous antithrombotic treatment options available, the combined use of multiple antithrombotic treatments could be hypothesized to provide long-term survival benefits.

This study aimed to examine whether an incremental long-term survival benefit could be observed by combined usage of a potent P2Y12 receptor inhibitor (ticagrelor instead of clopidogrel), GP IIb/IIIa inhibitor, and manual aspiration thrombectomy in STEMI patients treated with primary PCI.

## Materials and methods

### Study population

**FIGURE 1:**
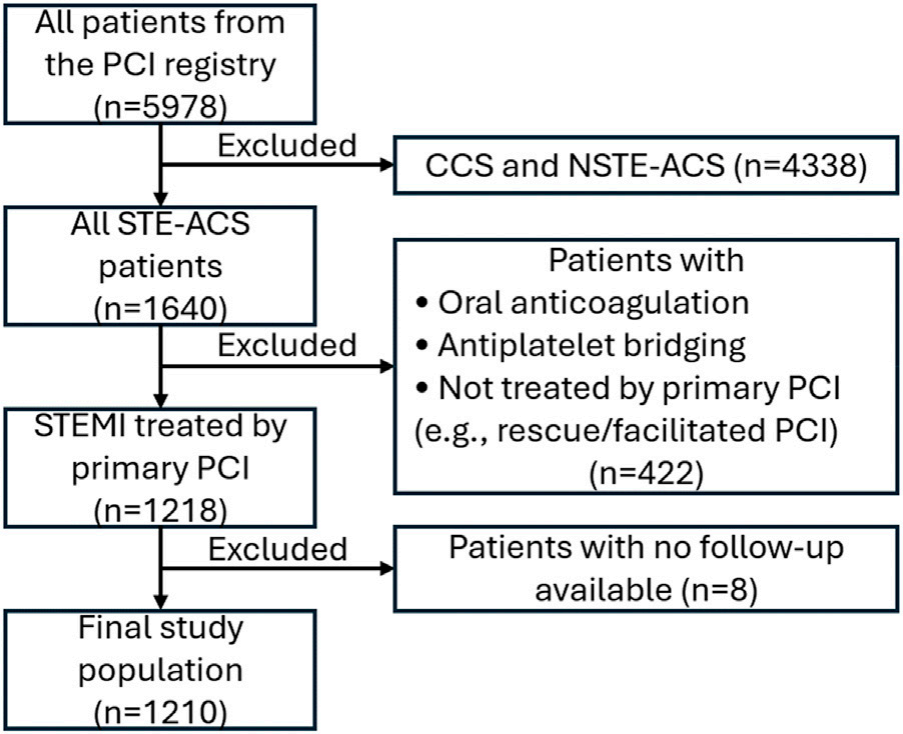
Flowchart of the present study. CCS, chronic coronary syndrome; NSTE-ACS, non-ST elevation acute coronary syndrome; PCI, percutaneous coronary intervention; STE-ACS, ST elevation acute coronary syndrome; STEMI, ST-segment elevation acute myocardial infarction.

All patients treated with PCI in the Emergency Institute for Cardiovascular Diseases and Transplantation of Târgu Mureş have been prospectively included at discharge in the local PCI registry of the institute since January 2016. Inclusion criteria for the current analysis consisted of 1) STEMI diagnosis treated with primary PCI by drug eluting stent angioplasty between January 2016 and December 2022, 2) dual antiplatelet therapy with aspirin and either clopidogrel or ticagrelor, and 3) periprocedural anticoagulation with unfractionated heparin. Use of ticagrelor or clopidogrel was at the discretion of the clinician, while use of eptifibatide or manual thrombus aspiration during primary PCI was at the discretion of the operator. Eptifibatide was administered intravenously as a bolus dose of 180 mcg/kg in cases of high thrombus burden. The bolus dose was followed by a continuous intravenous infusion of 2 mcg/kg/min up to 24 h only in the case of suboptimal PCI results. If the creatinine clearance was <50 mL/min, then the infusion rate was adjusted to 1 mcg/kg/min. Manual aspiration thrombectomy was performed with 6 French Export Advance coronary aspiration catheter (Medtronic Inc.). Exclusion criteria consisted of 1) age less than 18 years, 2) presence of a long-term anticoagulation indication (e.g., atrial fibrillation), 3) bridging between clopidogrel or ticagrelor during hospitalization, and 4) lack of available survival data (e.g., foreign patients). The study flowchart is illustrated in Figure 1. The registry is accessible online at the website http://pci.cardio.ro/, has been previously described (Călburean et al., 2022), and is based on the criteria of Cardiology Audit and Registration Data Standards (CARDS) developed by the Department of Health and Children, European Society of Cardiology, Irish Cardiac Society, and the European Commission (Flynn et al., 2005). The CARDS recommendations address data regarding demographics, relevant medical history and comorbid conditions, clinical status at hospital admission, PCI indication, affected and treated coronary artery segments, use of invasive diagnostic or therapeutic devices, procedural complications, and medical treatment during hospitalization and at discharge and in-hospital evolution. All the information available regarding all the variables proposed in that document was collected for every included patient.

All patients or their legal representatives provided a signed informed consent regarding the PCI procedure and participation in the study. The study was approved by the Ethical Committee of our institution (decision number 8646/22 December 2015). The protocol was carried out in accordance with the ethical principles for medical research involving human subjects as established by the Declaration of Helsinki, protecting the confidentiality of the personal information of the patients.

### Follow-up and clinical outcomes

The clinical endpoint of this study was the incidence of cardiovascular-cause and all-cause mortality. In-hospital mortality data were available from the PCI registry. The Romanian National Health Insurance System database supplied mortality rates as of July 2023 for all the patients. For patients who had died during follow-up, the Regional Statistics Office of the Romanian National Institute of Statistics supplied the exact date and cause of death according to the 10th revision of the International Classification of Diseases (ICD-10). If the cause of death was attributed to diseases of the circulatory system, then death was considered to be due to cardiovascular causes.

### Statistical analysis

A significance level α of 0.05 and a 95% confidence interval (CI) were considered. Continuous variables were evaluated for normal distribution using the Shapiro–Wilk test. Continuous variables were reported as mean ± standard deviation, and parametric distributions were compared using non-paired Student’s *t*-test, while nonparametric distributions were compared using the Mann–Whitney test. Categorical variables were reported as absolute and relative frequencies and compared using Fisher’s exact test. Univariate Cox proportional hazard regression was used to predict the association in the form of the hazard ratio (HR) between observed survival and a single independent categorical variable. Multivariate Cox regression models were constructed in a stepwise manner, where variables that reduced Akaike’s information criterion were added to the model (Bozdogan, 1987). Statistical analysis was performed using R version 4.1.1 and RStudio version 1.4.17.

## Results

A total of 1,210 patients were included in the present study. Of those patients, 832 (68.76%) were men, the mean age was 62.27 + 11.86 years, and mean BMI was 28.32 + 4.13 kg/m^2^. The complete clinical characteristics of the studied patients are reported in Table 1. During a median follow-up time of 2.78 (1.00–4.88) years, a total of 299 (24.7%) and 252 (20.8%) patients died of all-cause and cardiovascular-cause mortality, respectively. A total of 142 (11.7%) patients suffered in-hospital death, which was considered to be due to cardiovascular causes. All patients received aspirin and bolus intravenous unfractionated heparin, while primary PCI was always performed with drug-eluting stent implantation.

**TABLE 1:** Complete clinical characteristics of the studied population.

Parameter	All patients (*n* = 1,210)	All-cause mortality			Cardiovascular-cause mortality		
		HR	95% CI	*p*	HR	95% CI	*p*
Baseline characteristics							
Male sex	832 (68.76%)	0.57	0.45–0.72	<10^−5^	0.54	0.42–0.69	<10^−6^
Age (years)	62.27 ± 11.86	2.56	2.00–3.28	<10^−13^	2.43	1.86–3.17	<10^−10^
BMI (kg/m^2^)	28.32 ± 4.13	0.70	0.54–0.89	<10^−2^	0.62	0.47–0.83	<10^−2^
Hypertension	471 (38.93%)	1.01	0.80–1.27	0.96	0.97	0.76–1.25	0.83
Hypercholesterolemia	358 (29.59%)	0.68	0.53–0.88	<10^−2^	0.63	0.47–0.84	<10^−2^
Smoking status	539 (44.55%)	0.59	0.46–0.75	<10^−4^	0.53	0.41–0.69	<10^−5^
Diabetes mellitus	155 (12.81%)	1.23	0.91–1.66	0.18	1.08	0.76–1.52	0.68
Prior MI	102 (8.43%)	1.80	1.29–2.50	<10^−3^	1.81	1.26–2.58	<10^−2^
Prior CABG	14 (1.16%)	0.82	0.26–2.55	0.73	0.98	0.31–3.04	0.97
Prior PCI	120 (9.92%)	1.05	0.72–1.53	0.80	1.07	0.71–1.60	0.76
COPD	94 (7.77%)	2.33	1.69–3.20	<10^−6^	2.00	1.39–2.87	<10^−3^
Creatinine (mg/dL)	1.05 ± 0.6	2.78	2.20–3.51	<10^−17^	3.18	2.45–4.13	<10^−17^
CrCl <45 mL/min	62 (5.12%)	4.59	3.34–6.30	<10^−20^	4.54	3.22–6.39	<10^−17^
LVEF (%)	40.85 ± 7.25	0.30	0.23–0.40	<10^−16^	0.21	0.15–0.29	<10^−18^
LVEF ≤40%	626 (51.74%)	1.58	1.26–1.99	<10^−3^	1.83	1.42–2.37	<10^−5^
Killip class ≥4	38 (3.14%)	6.47	4.33–9.65	<10^−19^	6.87	4.56–10.37	<10^−19^
TIMI pre-PCI ≤1	866 (71.57%)	1.37	1.04–1.80	0.02	1.30	0.98–1.75	0.07
Procedural characteristics							
Ticagrelor	699 (57.77%)	0.27	0.21–0.34	<10^−24^	0.23	0.17–0.30	<10^−24^
Eptifibatide	412 (34.05%)	0.72	0.57–0.92	<10^−2^	0.68	0.52–0.89	<10^−2^
Manual thrombus aspiration	539 (44.55%)	1.06	0.84–1.33	0.63	0.99	0.77–1.27	0.93
Complete revascularization	393 (32.48%)	0.70	0.54–0.90	<10^−2^	0.73	0.56–0.97	0.03
Maximum stent diameter	3.2 ± 0.46	0.61	0.48–0.78	<10^−4^	0.56	0.43–0.74	<10^−4^
Total stent length	28.32 ± 14.04	1.07	0.85–1.35	0.58	1.04	0.81–1.34	0.76
Number of stents	1.34 ± 0.69	1.33	1.03–1.70	0.03	1.41	1.08–1.84	0.01
Segments treated	1.24 ± 0.55	1.61	1.25–2.07	<10^−3^	1.75	1.33–2.29	<10^−4^
Predilatation	342.0 (28.26%)	1.53	1.21–1.94	<10^−3^	1.58	1.22–2.04	<10^−3^
Post-dilatation	393.0 (32.48%)	1.04	0.82–1.32	0.73	1.01	0.78–1.31	0.94
TIMI post-PCI ≤2	113 (9.34%)	2.88	2.16–3.84	<10^−13^	3.06	2.26–4.15	<10^−13^

BMI, body mass index; CABG, coronary artery bypass graft; CI, confidence interval; CrCl, creatinine clearance; HR, hazard ratio; LVEF, left ventricular ejection fraction; MI, myocardial infarction; PCI, percutaneous coronary intervention; TIMI, thrombolysis in myocardial infarction.

**FIGURE 2:**
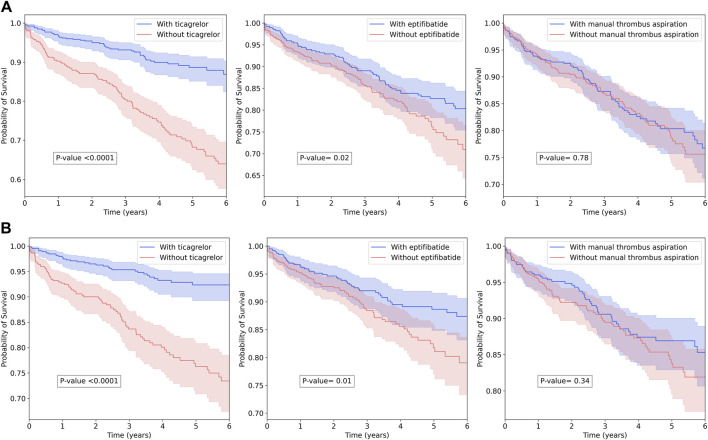
Kaplan–Meier plot and log-rank test for out-of-hospital survival among treatment groups. **(A)** All-cause mortality. **(B)** Cardiovascular-cause mortality.

**FIGURE 3:**
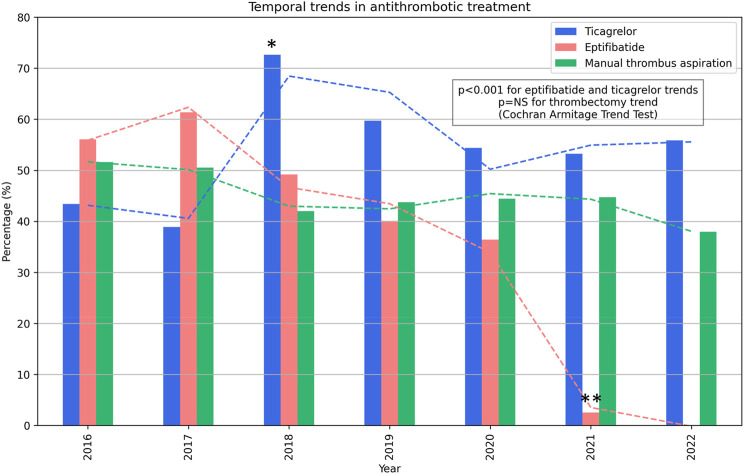
Temporal trends in the antithrombotic treatment use and observed survival. *A nationwide price-volume agreement for ticagrelor was implemented. **The nationwide price-volume agreement for eptifibatide expired and was not renewed.

Ticagrelor, the only potent P2Y12 inhibitor available, was administered to 699 (57.77%) patients, while clopidogrel was administered to the remaining 511 (42.23%) patients. Eptifibatide, the only GP IIb/IIIa inhibitor available, was administered to 412 (34.05%) patients, while manual thrombus aspiration was used for 539 (44.55%) patients. Ticagrelor significantly reduced all-cause mortality (HR = 0.27, 95% CI = 0.21–0.34, Cox *p* < 0.0001, and log-rank *p* < 0.0001) and cardiovascular-cause mortality (HR = 0.23, 95% CI = 0.17–0.30, Cox *p* < 0.0001, and log-rank *p* < 0.0001). Eptifibatide significantly reduced all-cause mortality (HR = 0.72, 95% CI = 0.57–0.92, Cox *p* = 0.002, and log-rank *p* = 0.02) and cardiovascular-cause mortality (HR = 0.68, 95% CI = 0.52–0.89, Cox *p* = 0.001, log-rank *p* = 0.01). Manual thrombus aspiration had no statistically significant effect on all-cause and cardiovascular-cause mortality. Kaplan–Meier curves are illustrated in Figure 2. There was a trend toward an increase in ticagrelor use, especially starting with 2019 when a nationwide price–volume agreement was established, and a peak of 70% use was achieved in 2019 (Figure 3). There was a trend toward a decrease in eptifibatide use, especially since 2020 when the nationwide price–volume agreement was not renewed, practically, eptifibatide was completely unavailable in 2021 and 2022 (Figure 3). There was no significant trend for manual thrombus aspiration use, and there was constant use during the inclusion period (Figure 3).

Patients receiving ticagrelor as a P2Y12 inhibitor instead of clopidogrel were more frequently men, of younger age, and with complete revascularization, while being less frequently with reduced LVEF, reduced creatinine clearance, or with post-PCI TIMI flow less than 3 (Table 2). Patients receiving eptifibatide were more frequently younger, diabetic, with pre-PCI TIMI flow ≤1, or with complete revascularization, while being less frequently with reduced LVEF or with post-PCI TIMI flow less than 3 (Table 2). Patients on whom manual thrombus aspiration was performed were more frequently younger, with pre-PCI TIMI flow ≤1 or with complete revascularization, while being less frequently with post-PCI TIMI flow less than 3 (Table 2). Baseline clinical characteristics comparisons between groups are reported in Tables 2, 3. Nearly all patients receiving all three therapies had thrombus-containing lesions as the pre-PCI TIMI flow was ≤1 in above 95% of the cases. Regarding bleeding risk, 10 (0.8%) patients suffered major clinical overt bleeding, 25 (2.0%) patients had a hemoglobin drop ≥5 g/dL after PCI, and 152 (12.5%) patients had a hemoglobin drop ≥15% after PCI, but there were no significant differences among treatment groups. No in-hospital death was attributed to bleeding events. A total of 1,098 (90.7%) of the procedures were performed by radial vascular access.

**TABLE 2:** Comparison of clinical characteristics between investigated pharmacological and mechanical therapies.

Parameter	Ticagrelor^a^			Eptifibatide			Manual thrombus aspiration		
	Without (*n* = 511)	With (*n* = 699)	*p*	Without (*n* = 798)	With (*n* = 412)	*p*	Without (*n* = 671)	With (*n* = 539)	*p*
Baseline characteristics									
Male sex	319 (62.43%)	513 (73.39%)	<0.001	537 (67.29%)	295 (71.6%)	0.12	454 (67.66%)	378 (70.13%)	0.40
Age (years)	66.2 (58.5–75.6)	60.0 (51.1–67.0)	<0.001	63.4 (53.8–72.5)	60.09 ± 11.21	<0.001	63.1 (53.8–71.6)	61.05 ± 11.88	0.002
BMI (kg/m^2^)	27.7 (25.8–29.3)	27.7 (26.5–30.0)	0.04	27.7 (26.7–29.3)	27.7 (25.7–30.5)	0.74	27.7 (26.2–29.4)	27.7 (26.1–30.0)	0.98
Hypertension	208 (40.7%)	263 (37.63%)	0.27	250 (31.33%)	221 (53.64%)	<0.001	251 (37.41%)	220 (40.82%)	0.24
Hypercholesterolemia	136 (26.61%)	222 (31.76%)	0.05	177 (22.18%)	181 (43.93%)	<0.001	205 (30.55%)	153 (28.39%)	0.42
Smoking status	196 (38.36%)	343 (49.07%)	<0.001	333 (41.73%)	206 (50%)	0.007	277 (41.28%)	262 (48.61%)	0.01
Diabetes mellitus	72 (14.09%)	83 (11.87%)	0.24	84 (10.53%)	71 (17.23%)	0.001	96 (14.31%)	59 (10.95%)	0.08
Prior MI	59 (11.55%)	43 (6.15%)	0.002	66 (8.27%)	36 (8.74%)	0.83	59 (8.79%)	43 (7.98%)	0.68
Prior PCI	57 (11.15%)	63 (9.01%)	0.25	79 (9.9%)	41 (9.95%)	0.99	59 (8.79%)	61 (11.32%)	0.14
Prior CABG	6 (1.17%)	8 (1.14%)	0.99	9 (1.13%)	5 (1.21%)	0.99	8 (1.19%)	6 (1.11%)	0.99
COPD	49 (9.59%)	45 (6.44%)	0.04	61 (7.64%)	33 (8.01%)	0.90	60 (8.94%)	34 (6.31%)	0.09
Creatinine (mg/dL)	0.92 (0.79–1.21)	0.87 (0.78–1.04)	<0.001	0.89 (0.78–1.1)	0.88 (0.78–1.08)	0.70	0.89 (0.78–1.08)	0.89 (0.78–1.11)	0.95
CrCl <45 mL/min	40 (7.83%)	22 (3.15%)	<0.001	39 (4.89%)	23 (5.58%)	0.68	33 (4.92%)	29 (5.38%)	0.79
LVEF (%)	40 (35–45)	40 (40–45)	<0.001	40 (35–45)	40 (40–45)	<0.001	40 (35–45)	40 (40–45)	0.09
LVEF ≤40%	294 (57.53%)	332 (47.5%)	0.001	428 (53.63%)	198 (48.06%)	0.06	358 (53.35%)	268 (49.72%)	0.23
Killip class ≥4	30 (5.87%)	8 (1.14%)	<0.001	30 (3.76%)	8 (1.94%)	0.12	16 (2.38%)	22 (4.08%)	0.09
TIMI pre-PCI ≤1	350 (68.49%)	516 (73.82%)	0.04	504 (63.16%)	362 (87.86%)	<0.001	379 (56.48%)	487 (90.35%)	<0.001
Procedural characteristics									
Complete revascularization	135 (26.42%)	258 (36.91%)	<0.001	231 (28.95%)	162 (39.32%)	<0.001	195 (29.06%)	198 (36.73%)	0.006
Maximum stent diameter	3 (3–3.5)	3 (3–3.5)	0.009	3 (3–3.5)	3 (3–3.5)	0.18	3 (3–3.5)	3 (3–3.5)	<0.001
Total stent length	26 (18–32)	26 (18–32)	0.59	26 (18–32)	26 (18–30)	0.84	26 (18–30)	26 (18–33)	0.53
Number of stents	1 (1–2)	1 (1–1)	0.42	1 (1–1)	1 (1–2)	0.41	1 (1–2)	1 (1–1)	0.09
Segments treated	1 (1–1)	1 (1–1)	0.72	1 (1–1)	1 (1–1)	0.72	1 (1–1)	1 (1–1)	0.12
Predilatation	168 (32.88%)	174 (24.89%)	0.002	229 (28.7%)	113 (27.43%)	0.69	225 (33.53%)	117 (21.71%)	<0.001
Post-dilatation	168 (32.88%)	225 (32.19%)	0.81	257 (32.21%)	136 (33.01%)	0.79	236 (35.17%)	157 (29.13%)	0.02
TIMI post-PCI ≤2	69 (13.5%)	44 (6.29%)	<0.001	62 (7.77%)	51 (12.38%)	0.01	47 (7%)	66 (12.24%)	0.002

^a^ Without ticagrelor, implying that clopidogrel was administered instead. BMI, body mass index; CABG, coronary artery bypass graft; CrCl, creatinine clearance; LVEF, left ventricular ejection fraction; MI, myocardial infarction; PCI, percutaneous coronary intervention; TIMI, thrombolysis in myocardial infarction.

**TABLE 3:** Comparison of clinical characteristics between treatment groups.

Parameter	Clopidogrel only (*n* = 239)	Ticagrelor only (*n* = 281)	Eptifibatide only (*n* = 57)	Aspiration only (*n* = 125)	Ticagrelor + eptifibatide (*n* = 94)	Ticagrelor + aspiration (*n* = 153)	Eptifibatide + aspiration (*n* = 90)	All therapies^a^ (*n* = 171)	*p*
Baseline characteristics									
Male sex	143 (59.8%)	199 (70.8%)	40 (70.2%)	81 (64.8%)	72 (76.6%)	114 (74.5%)	55 (61.1%)	128 (74.9%)	<10^−2^
Age (years)	65.1 + 13.1	60.0 + 11.4	67.9 + 11.9	57.7 + 10.8	60.7 + 10.6	57.5 + 10.4	63.9 + 11.3	65.8 + 10.5	<10^−21^
BMI (kg/m^2^)	27.9 + 4.1	28.8 + 4.1	28.2 + 4.3	28.5 + 3.8	28.3 + 3.8	28.7 + 4.4	27.9 + 4.1	28.0 + 4.7	0.54
Hypertension	86 (36.0%)	86 (30.6%)	32 (56.1%)	41 (32.8%)	47 (50.0%)	37 (24.2%)	49 (54.4%)	93 (54.4%)	<10^−10^
Hypercholesterolemia	54 (22.6%)	83 (29.5%)	24 (42.1%)	18 (14.4%)	44 (46.8%)	22 (14.4%)	40 (44.4%)	73 (42.7%)	<10^−13^
Smoking status	80 (33.5%)	132 (47.0%)	22 (38.6%)	51 (40.8%)	43 (45.7%)	70 (45.8%)	43 (47.8%)	98 (57.3%)	<10^−3^
Diabetes mellitus	32 (13.4%)	36 (12.8%)	13 (22.8%)	11 (8.8%)	15 (16.0%)	5 (3.3%)	16 (17.8%)	27 (15.8%)	<10^−2^
Prior MI	30 (12.6%)	17 (6.0%)	9 (15.8%)	9 (7.2%)	3 (3.2%)	10 (6.5%)	11 (12.2%)	13 (7.6%)	0.01
Prior PCI	3 (1.3%)	2 (0.7%)	1 (1.8%)	1 (0.8%)	2 (2.1%)	3 (2.0%)	1 (1.1%)	1 (0.6%)	0.89
Prior CABG	24 (10.0%)	24 (8.5%)	6 (10.5%)	17 (13.6%)	5 (5.3%)	14 (9.2%)	10 (11.1%)	20 (11.7%)	0.59
COPD	24 (10.0%)	24 (8.5%)	4 (7.0%)	6 (4.8%)	8 (8.5%)	7 (4.6%)	15 (16.7%)	6 (3.5%)	<10^−^_2_
Creatinine (mg/dL)	1.3 + 1.3	1.0 + 0.4	1.1 + 0.7	1.0 + 0.4	0.9 + 0.2	0.9 + 0.3	1.1 + 0.5	1.1 + 0.5	<10^−2^
CrCl <45 mL/min	15 (6.3%)	6 (2.1%)	6 (10.5%)	12 (9.6%)	6 (6.4%)	6 (3.9%)	7 (7.8%)	4 (2.3%)	<10^−2^
LVEF (%)	39.5 + 8.0	41.5 + 5.6	39.1 + 7.2	43.2 + 7.5	41.4 + 6.3	42.8 + 7.5	40.0 + 8.4	38.8 + 7.9	<10^−5^
LVEF ≤40%	139 (58.2%)	145 (51.6%)	36 (63.2%)	72 (57.6%)	38 (40.4%)	72 (47.1%)	47 (52.2%)	77 (45.0%)	0.01
Killip class ≥4	10.0 (4.2%)	4.0 (1.4%)	2.0 (3.5%)	13.0 (10.4%)	0.0 (0.0%)	3.0 (2.0%)	5.0 (5.6%)	1.0 (0.6%)	<10^−4^
TIMI pre-PCI ≤1	114 (47.7%)	149 (53.0%)	44 (77.2%)	109 (87.2%)	72 (76.6%)	132 (86.3%)	83 (92.2%)	163 (95.3%)	<10^−41^
Procedural characteristics									
Complete revascularization	53 (22.2%)	93 (33.1%)	14 (24.6%)	33 (26.4%)	35 (37.2%)	52 (34.0%)	35 (38.9%)	78 (45.6%)	<10^−4^
Maximum stent diameter	3.2 + 0.4	3.2 + 0.5	3.1 + 0.4	3.1 + 0.4	3.2 + 0.5	3.3 + 0.5	3.2 + 0.4	3.1 + 0.5	<10^−4^
Total stent length	28.5 + 17.2	29.5 + 16.4	29.5 + 14.4	27.3 + 12.6	26.9 + 12.1	29.5 + 14.0	26.8 + 10.2	27.7 + 14.0	0.63
Number of stents	1.3 + 0.8	1.3 + 0.7	1.4 + 0.7	1.4 + 0.7	1.3 + 0.6	1.3 + 0.7	1.3 + 0.5	1.5 + 0.9	0.37
Segments treated	1.2 + 0.5	1.2 + 0.6	1.3 + 0.6	1.2 + 0.4	1.2 + 0.5	1.2 + 0.5	1.1 + 0.4	1.5 + 1.0	0.19
Predilatation	95 (39.7%)	78 (27.8%)	25 (43.9%)	26 (20.8%)	27 (28.7%)	30 (19.6%)	22 (24.4%)	39 (22.8%)	<10^−4^
Post-dilatation	90 (37.7%)	91 (32.4%)	18 (31.6%)	33 (26.4%)	37 (39.4%)	43 (28.1%)	27 (30.0%)	54 (31.6%)	0.27
TIMI post-PCI ≤2	25 (10.5%)	10 (3.6%)	7 (12.3%)	20 (16.0%)	5 (5.3%)	7 (4.6%)	17 (18.9%)	22 (12.9%)	<10^−5^

^a^ All therapies refer to concomitant ticagrelor, eptifibatide, and manual thrombus aspiration use.

BMI, body mass index; CABG, coronary artery bypass graft; CrCl, creatinine clearance; LVEF, left ventricular ejection fraction; MI, myocardial infarction; PCI, percutaneous coronary intervention; TIMI, thrombolysis in myocardial infarction.

**FIGURE 4:**
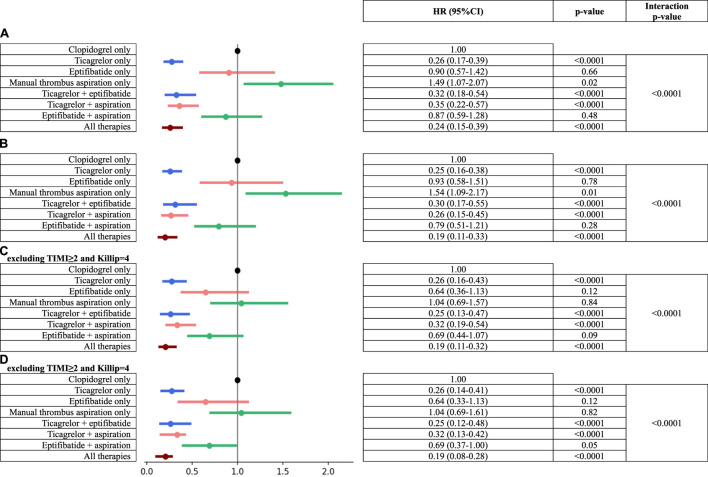
Risk of adverse events among treatment groups. **(A)** All-cause mortality. **(B)** Cardiovascular-cause mortality. **(C)** All-cause mortality excluding TIMI ≥2 and Killip = 4. **(D)** Cardiovascular-cause mortality excluding TIMI ≥2 and Killip = 4.

When comparing survival among different treatment groups, it was observed that ticagrelor-only and ticagrelor-containing groups had significantly better cardiovascular and all-cause survival than the reference group (clopidogrel-only group, Figures 4A–D). The manual thrombus aspiration-alone group (with clopidogrel as a P2Y12 inhibitor) had significantly worse cardiovascular and all-cause survival than the reference group (clopidogrel-only group, Figures 4A, B). Eptifibatide alone and eptifibatide with manual thrombus aspiration groups had no effect on cardiovascular and all-cause survival in comparison with the reference group (clopidogrel-only group, Figures 4A, B). Interestingly, in patients with pre-PCI TIMI flow ≤1 and without Killip class IV, the combined use of eptifibatide and manual thrombus aspiration was associated with reduced cardiovascular-cause mortality, while eptifibatide alone or manual thrombus aspiration alone did not impact survival (Figures 4C, D). Moreover, survival in the ticagrelor-only group was similar to that in the other groups containing ticagrelor (ticagrelor and eptifibatide, ticagrelor and manual thrombus aspiration, and all therapies groups), revealing that eptifibatide and manual thrombus aspiration did not have additional survival benefits.

**FIGURE 5:**
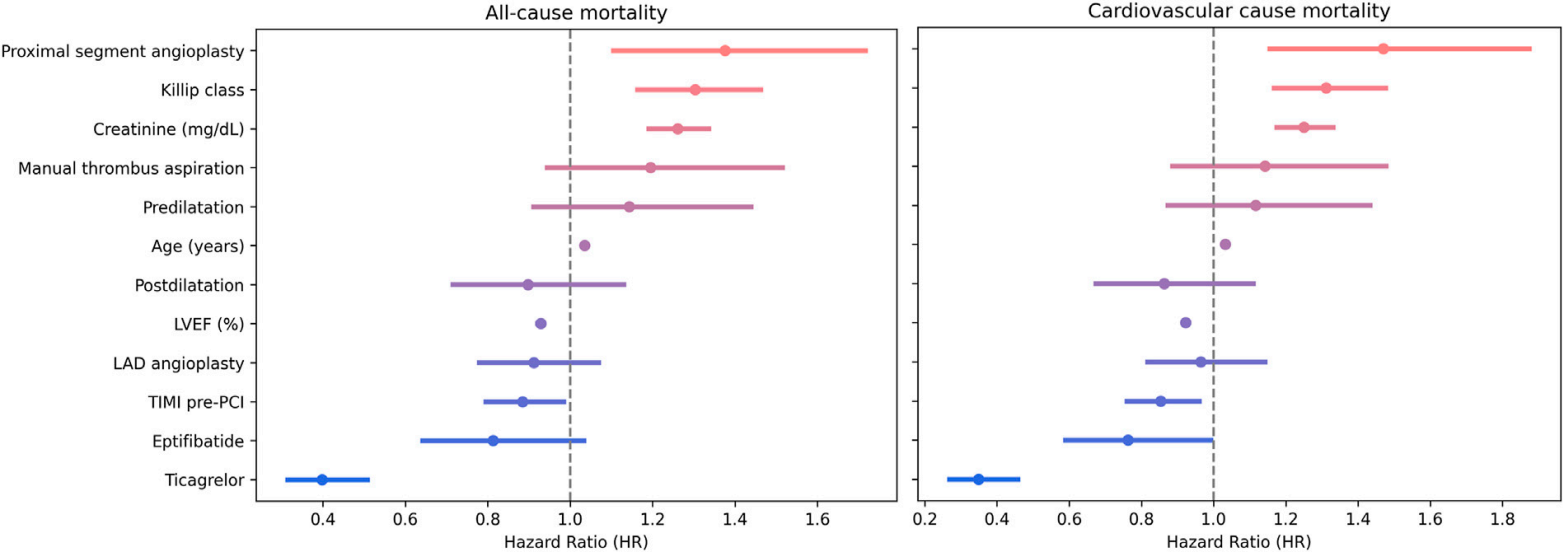
Multivariate Cox proportional hazards for mortality prediction.

When adjusting for potential confounders in a Cox multivariate regression, the presence of ticagrelor, higher pre-PCI TIMI flow, or higher LVEF were protective against all-cause mortality; older age, higher creatinine, higher Killip class, or the presence of proximal segment culprit lesions predisposed for all-cause mortality, while eptifibatide or manual thrombus aspiration did not affect survival. Similarly, the presence of ticagrelor, administration of eptifibatide, higher pre-PCI TIMI flow, or higher LVEF were protective factors against cardiovascular-cause mortality, and older age, higher creatinine, higher Killip class, or the presence of proximal segment culprit lesion predisposed for cardiovascular-cause mortality, while manual thrombus aspiration did not affect survival (Figure 5).

## Discussions

Our findings can be summarized as follows: 1) ticagrelor consistently offered survival benefits in all performed analyses, including multivariate Cox regression; 2) eptifibatide had a protective effect against cardiovascular-cause mortality, but not for all-cause mortality in multivariate Cox regression; 3) manual thrombus aspiration had no effect on survival; 4) the only survival benefit of manual thrombus aspiration was observed in eptifibatide and clopidogrel combination therapy when compared with the clopidogrel-only group in patients with pre-PCI TIMI flow ≤1 and without cardiogenic shock; 5) ticagrelor survival benefit was independent of eptifibatide and manual thrombus aspiration; moreover, the addition of eptifibatide or manual thrombus aspiration to ticagrelor did not lead to an improved outcome.

Over the recent decades, numerous antithrombotic treatments have been investigated in STEMI patients. The benefit of mechanical reperfusion through primary PCI with drug-eluting stent and rapid platelet inhibition by DAPT with aspirin and a potent P2Y12 receptor inhibitor (ticagrelor/prasugrel instead of clopidogrel) is undisputable and is the standard of care. It is unknown whether other antithrombotic options could add incremental survival benefits. The current era of potent P2Y12 inhibitors began with PLATO and TRINITON-TIMI 38 studies that showed significantly reduced cardiovascular and all-cause death in STEMI with ticagrelor and prasugrel, respectively, in comparison with clopidogrel (Montalescot et al., 2009; Steg et al., 2010). Before DAPT, GP IIb/IIIa inhibitors were routinely administered during primary PCI (De Luca et al., 2005). After the introduction of DAPT with clopidogrel and stent angioplasty, GP IIb/IIIa benefit decreased, and it became less used (Brener et al., 1998; Neumann et al., 2000; Montalescot et al., 2001; Stone et al., 2002). Nevertheless, GP IIb/IIIa inhibitors were administered in 35% and 63% of STEMI patients from PLATO and TRINITON-TIMI 38 studies. Interestingly, the survival benefit of ticagrelor in the STEMI subgroup from the PLATO study was lower in patients with additional GP IIb/IIIa inhibitors than in patients without additional GP IIb/IIIa inhibitors (Steg et al., 2010), while a meta-analysis showed improved survival benefits with ticagrelor or prasugrel than with clopidogrel in STEMI patients undergoing primary PCI and receiving aspirin and GP IIb/IIIa inhibitors (Wang et al., 2020). This shows that while DAPT with potent P2Y12 clearly reduces incidences of adverse outcomes, additional GP IIb/IIIa inhibitors could also additionally reduce adverse outcomes, at least in certain subgroups. In our study, eptifibatide significantly reduced cardiovascular and all-cause mortality on log-rank and univariate Cox regression (Table 1; Figure 2), while it did not reduce all-cause mortality and only borderline reduced cardiovascular mortality (*p* = 0.05) in multivariable analysis (Figure 4). Even though it was stated to be unclear, we showed that ticagrelor benefit is independent of GP IIb/IIIa inhibitors, even in the context of pre-PCI TIMI flow ≤1. A possible explanation could be that ticagrelor has a protective effect against ischemia independently of its antiplatelet effects. A recent study demonstrated ticagrelor-associated prevention of human endothelial cell apoptosis in the early stages of hypoxia through the adenosine signaling pathway (Feliu et al., 2020). A meta-analysis found that ticagrelor significantly improved endothelial function in comparison with prasugrel, clopidogrel, and placebo, increasing flow-mediated vasodilation, the reactive hyperemia index, and the number of circulating endothelial progenitor cells, while reducing the index of coronary microvascular resistance (Guan et al., 2022). Moreover, ticagrelor could improve blood rheology (Rosenson et al., 2019), which was repeatedly shown to affect clinical outcomes in STEMI (Kaplangoray et al., 2023a; Kaplangoray et al., 2023b; Kaplangoray et al., 2023c).

Manual thrombus aspiration implies mechanical removal of coronary blood clot and is an attractive option for antithrombotic therapy since it is not associated with increased bleeding risk as in the case of pharmacologic treatment. Initial small studies reported short-term clinical benefits that failed to be reproduced in large, randomized trials, and a surprising risk of stroke was observed (Bavry et al., 2008; Deng et al., 2014; El Dib et al., 2016). Importantly, manual thrombus aspiration is a safe option even in the clinical context of elderly or frail patients (Mone et al., 2021). In our study, manual thrombus aspiration was observed to have no effect on survival—the only survival benefit of manual thrombus aspiration was observed in the combination therapy of eptifibatide and clopidogrel when compared with the clopidogrel-only group in thrombus-containing lesions (pre-PCI TIMI flow ≤1) and without cardiogenic shock. Our findings support the current guideline recommendation that eptifibatide and manual thrombus aspiration should not be routinely used in STEMI patients treated with primary PCI, but only in cases with high thrombotic burden. Notably, thrombus aspiration was used only selectively in our center after 2015, according to the guideline recommendations based on landmark studies (Bavry et al., 2008; Deng et al., 2014; El Dib et al., 2016). Based on our results, a pre-PCI TIMI score ≤1 seems insufficient to reflect a high thrombotic burden and justify the use of manual thrombus aspiration and/or eptifibatide. There are other angiographic scores that presumably better quantify thrombotic burden than the TIMI score, such as the TIMI thrombus score or the TIMI frame count, but they may prolong the time to stent expansion (Gibson et al., 1996; Napodano et al., 2014). While a more intensive approach for antithrombotic treatment would seem logical, with the lack of significant evidence, current guideline recommendations should not be deemed defensive (Rakowski et al., 2023). Intramyocardial hemorrhage is a known complication of intensive antithrombotic medication, such as GP IIb/IIIa, and is significantly associated with impaired survival after STEMI (Amier et al., 2017; Vyas et al., 2022). Regarding safety outcomes, less than 1% patients suffered major clinical bleeding, and no in-hospital deaths were attributed to bleeding events. This may be due to the exclusion criteria that are known to predispose to bleeding risk (e.g., the presence of an oral anticoagulation indication, bridging between antiplatelet agents, and no thrombolytics were used since only primary PCI procedures were considered) and the high use of radial vascular access (femoral arterial access also predispose to risk of hemorrhagic events).

## Study limitations

Lack of data regarding more precise thrombotic burden quantification methods [e.g., TIMI frame count (Gibson et al., 1996)] regarding myocardial perfusion scores [e.g., myocardial blush grade (Seyfeli et al., 2007; Porto et al., 2010)] or details regarding successful aspiration (e.g., visible removal of thrombotic material) could miss a significant effect of manual thrombus aspiration on survival in certain subgroups. In addition, the study population is typical of Eastern Europe, consisting of exclusively white individuals; thus, extrapolating the results to other populations could be limited.

## Conclusion

In STEMI patients treated with primary PCI, ticagrelor consistently reduced cardiovascular and all-cause mortality on all performed analyses, and the benefit is independent from GP IIb/IIIa inhibitors and manual thrombus aspiration, even in the context of pre-PCI TIMI flow ≤1. GP IIb/IIIa inhibitors marginally reduced cardiovascular mortality, while manual thrombus aspiration did not impact survival. The only potential benefit of manual thrombus aspiration would be when given in combination with clopidogrel and GP IIb/IIIa inhibitors in pre-PCI TIMI flow ≤1. Our findings support the current guideline recommendation that eptifibatide and manual thrombus aspiration should not be routinely used in STEMI patients treated with primary PCI.

## Data Availability

The raw data supporting the conclusion of this article will be made available by the authors, without undue reservation.

## References

[B1] Amier R. P. Tijssen R. Y. G. Teunissen P. F. A. Fernández-Jiménez R. Pizarro G. García-Lunar I. (2017). Predictors of intramyocardial hemorrhage after reperfused ST‐segment elevation myocardial infarction. J. Am. Heart Assoc. 6, e005651. 10.1161/JAHA.117.005651 28862937 PMC5586425

[B2] Bavry A. A. Kumbhani D. J. Bhatt D. L. (2008). Role of adjunctive thrombectomy and embolic protection devices in acute myocardial infarction: a comprehensive meta-analysis of randomized trials. Eur. Heart J. 29, 2989–3001. 10.1093/eurheartj/ehn421 18812323

[B3] Bozdogan H. (1987). Model selection and Akaike’s Information Criterion (AIC): the general theory and its analytical extensions. Psychometrika 52, 345–370. 10.1007/bf02294361

[B4] Brener S. J. Barr L. A. Burchenal J. E. Katz S. George B. S. Jones A. A. (1998). Randomized, placebo-controlled trial of platelet glycoprotein IIb/IIIa blockade with primary angioplasty for acute myocardial infarction. ReoPro and Primary PTCA Organization and Randomized Trial (RAPPORT) Investigators. Circulation 98, 734–741. 10.1161/01.cir.98.8.734 9727542

[B5] Byrne R. A. (2023). 2023 ESC Guidelines for the management of acute coronary syndromes: developed by the task force on the management of acute coronary syndromes of the European Society of Cardiology (ESC). Eur. Heart J. ehad, 1 91. 10.1093/eurheartj/ehad191 38383069

[B6] Călburean P.-A. Grebenișan P. Nistor I. A. Pal K. Vacariu V. Drincal R. K. (2022). Prediction of 3-year all-cause and cardiovascular cause mortality in a prospective percutaneous coronary intervention registry: machine learning model outperforms conventional clinical risk scores. Atherosclerosis 350, 33–40. 10.1016/j.atherosclerosis.2022.03.028 35483116

[B7] De Luca G. Suryapranata H. Stone G. W. Antoniucci D. Tcheng J. E. Neumann F. J. (2005). Abciximab as adjunctive therapy to reperfusion in acute ST-segment elevation myocardial infarction: a meta-analysis of randomized trials. JAMA 293, 1759–1765. 10.1001/jama.293.14.1759 15827315

[B8] Deng S.-B. Wang J. Xiao J. Wu L. Jing X. D. Yan Y. L. (2014). Adjunctive manual thrombus aspiration during ST-segment elevation myocardial infarction: a meta-analysis of randomized controlled trials. PLoS One 9, e113481. 10.1371/journal.pone.0113481 25405874 PMC4236171

[B9] El Dib R. Spencer F. A. Suzumura E. A. Gomaa H. Kwong J. Guyatt G. H. (2016). Aspiration thrombectomy prior to percutaneous coronary intervention in ST-elevation myocardial infarction: a systematic review and meta-analysis. BMC Cardiovasc. Disord. 16, 121. 10.1186/s12872-016-0285-4 27255331 PMC4890469

[B10] Feliu C. Peyret H. Brassart-Pasco S. Oszust F. Poitevin G. Nguyen P. (2020). Ticagrelor prevents endothelial cell apoptosis through the adenosine signalling pathway in the early stages of hypoxia. Biomolecules 10, 740. 10.3390/biom10050740 32397519 PMC7277469

[B11] Flynn M. R. Barrett C. Cosío F. G. Gitt A. K. Wallentin L. Kearney P. (2005). The Cardiology Audit and Registration Data Standards (CARDS), European data standards for clinical cardiology practice. Eur. Heart J. 26, 308–313. 10.1093/eurheartj/ehi079 15618029

[B12] Gibson C. M. Cannon C. P. Daley W. L. Dodge J. T. Alexander B. Marble S. J. (1996). TIMI frame count: a quantitative method of assessing coronary artery flow. Circulation 93, 879–888. 10.1161/01.cir.93.5.879 8598078

[B13] Guan B. Zhao L. Ma D. Fan Y. Zhang H. Wang A. (2022). The effect of ticagrelor on endothelial function compared to prasugrel, clopidogrel, and placebo: a systematic review and meta-analysis. Front. Cardiovasc. Med. 8, 820604. 10.3389/fcvm.2021.820604 35155620 PMC8826068

[B14] Kaplangoray M. Toprak K. Aslan R. Deveci E. Gunes A. Ardahanli İ. (2023a). High CRP-albumin ratio is associated high thrombus burden in patients with newly diagnosed STEMI. Medicine 102, e35363. 10.1097/MD.0000000000035363 37832116 PMC10578711

[B15] Kaplangoray M. Toprak K. Cekici Y. Yildirim A. Abacioglu O. O. (2023c). Relationship between blood viscosity and thrombus burden in ST-segment elevation myocardial infarction. Clin. Hemorheol. Microcirc. 85, 31–40. 10.3233/CH-231756 37522201

[B16] Kaplangoray M. Toprak K. Cicek O. F. Deveci E. (2023b). Relationship between the fibrinogen/albumin ratio and microvascular perfusion in patients undergoing primary percutaneous coronary intervention for ST-elevated myocardial infarction: a prospective study. Arq. Bras. Cardiol. 120, e20230002. 10.36660/abc.20230002 38661580 PMC12087635

[B17] Mone P. Gambardella J. Pansini A. Rizzo M. Mauro C. Minicucci F. (2021). Impact of thrombus aspiration in frail STEMI patients. Aging Clin. Exp. Res. 33, 3081–3089. 10.1007/s40520-021-01848-5 33817772 PMC8488061

[B18] Montalescot G. Barragan P. Wittenberg O. Ecollan P. Elhadad S. Villain P. (2001). Platelet glycoprotein IIb/IIIa inhibition with coronary stenting for acute myocardial infarction. N. Engl. J. Med. 344, 1895–1903. 10.1056/NEJM200106213442503 11419426

[B19] Montalescot G. Wiviott S. D. Braunwald E. Murphy S. A. Gibson C. M. McCabe C. H. (2009). Prasugrel compared with clopidogrel in patients undergoing percutaneous coronary intervention for ST-elevation myocardial infarction (TRITON-TIMI 38): double-blind, randomised controlled trial. Lancet 373, 723–731. 10.1016/S0140-6736(09)60441-4 19249633

[B20] Napodano M. Dariol G. Al Mamary A. H. Marra M. P. Tarantini G. D'Amico G. (2014). Thrombus burden and myocardial damage during primary percutaneous coronary intervention. Am. J. Cardiol. 113, 1449–1456. 10.1016/j.amjcard.2014.01.423 24630783

[B21] Neumann F.-J. Kastrati A. Schmitt C. Blasini R. Hadamitzky M. Mehilli J. (2000). Effect of glycoprotein IIb/IIIa receptor blockade with abciximab on clinical and angiographic restenosis rate after the placement of coronary stents following acute myocardial infarction. J. Am. Coll. Cardiol. 35, 915–921. 10.1016/s0735-1097(99)00635-x 10732888

[B22] Porto I. Hamilton-Craig C. Brancati M. Burzotta F. Galiuto L. Crea F. (2010). Angiographic assessment of microvascular perfusion—myocardial blush in clinical practice. Am. Heart J. 160, 1015–1022. 10.1016/j.ahj.2010.08.009 21146652

[B23] Rakowski T. Węgiel M. Malinowski K. P. Siudak Z. Zasada W. Zdzierak B. (2023). Thrombus containing lesions strategies during primary percutaneous coronary interventions in ST-segment elevation myocardial infarction: insights from ORPKI National Registry. J. Thromb. Thrombolysis 56, 156–163. 10.1007/s11239-023-02811-z 37093352 PMC10284931

[B24] Rosenson R. S. Chen Q. Najera S. D. Krishnan P. Lee M. L. Cho D. J. (2019). Ticagrelor improves blood viscosity-dependent microcirculatory flow in patients with lower extremity arterial disease: the Hema-kinesis clinical trial. Cardiovasc Diabetol. 18, 77. 10.1186/s12933-019-0882-5 31174526 PMC6556022

[B25] Seyfeli E. Abaci A. Kula M. Topsakal R. Eryol N. K. Arinc H. (2007). Myocardial blush grade: to evaluate myocardial viability in patients with acute myocardial infarction. Angiology 58, 556–560. 10.1177/0003319707307846 18024938

[B26] Steg P. G. James S. Harrington R. A. Ardissino D. Becker R. C. Cannon C. P. (2010). Ticagrelor versus clopidogrel in patients with ST-elevation acute coronary syndromes intended for reperfusion with primary percutaneous coronary intervention: a Platelet Inhibition and Patient Outcomes (PLATO) trial subgroup analysis. Circulation 122, 2131–2141. 10.1161/CIRCULATIONAHA.109.927582 21060072

[B27] Stone G. W. Grines C. L. Cox D. A. Garcia E. Tcheng J. E. Griffin J. J. (2002). Comparison of angioplasty with stenting, with or without abciximab, in acute myocardial infarction. N. Engl. J. Med. 346, 957–966. 10.1056/NEJMoa013404 11919304

[B28] Vyas R. Changal K. H. Bhuta S. Pasadyn V. Katterle K. Niedoba M. J. (2022). Impact of intramyocardial hemorrhage on clinical outcomes in ST-elevation myocardial infarction: a systematic review and meta-analysis. J. Soc. Cardiovasc. Angiogr. Interventions 1, 100444. 10.1016/j.jscai.2022.100444 PMC11307811 39132339

[B29] Wang Z. Zhou D.-Y. Su Y. Si L.-Y. Xu Q. (2020). Prasugrel or ticagrelor relative to clopidogrel in triple-antiplatelet treatment combined with glycoprotein IIb/IIIa inhibitor for patients with STEMI undergoing PCI: a meta-analysis. BMC Cardiovasc. Disord. 20, 130. 10.1186/s12872-020-01403-6 32164560 PMC7066764

